# Use of Bariatric Ports in 4-Arm Robotic Partial Nephrectomy: A Comparative Study With the Standard 3-Arm Technique

**DOI:** 10.7759/cureus.16461

**Published:** 2021-07-18

**Authors:** Jose M El-Asmar, Ralph Sebaaly, Aurelie Mailhac, Muhammad Bulbul, Raja Khauli, Hani Tamim, Albert El Hajj

**Affiliations:** 1 Department of Urology, American University of Beirut Medical Center, Beirut, LBN

**Keywords:** partial nephrectomy, robotics, fourth arm, ports, renal cell carcinoma

## Abstract

Objectives

We aim to compare the outcomes of a 3-arm versus a 4-arm robotic assisted partial nephrectomy (RAPN) using the da Vinci Si model; as well as, illustrate the deployment of long ports to decrease arm collision during the 4-arm approach.

Patients and Methods

Results of RAPN in a Middle Eastern tertiary referral center from August 2013 to December 2017 are reported. Comparison between 3 versus 4-arm robotic approaches was done in regards to patient and tumor characteristics, operative parameters, and postoperative outcomes. Statistical analysis was performed with the Student’s t-test and chi-squared test.

Results

Forty consecutive 3-arm RAPNs and 40 consecutive 4-arm RAPNs were retrospectively evaluated. Differences in tumor complexity between the two groups were statistically insignificant. Similarly, surgical margin positivity, mean ischemia time, estimated blood loss, length of hospital stay, and mean change in serum creatinine were statistically insignificant between the two groups. Mean operative time was significantly shorter by 42 minutes in the 4-arm vs 3-arm group (p=0.01).

Conclusions

The addition of a 4^th^ arm in RAPN can be of benefit in centers that still rely on the da Vinci Si model. The ease of hilar dissection, retraction, and surgeon independence instigated a statistically significant decrease in operative time with 4-arm use.

## Introduction

Open, laparoscopic, or robotic assisted partial nephrectomy is the surgical procedure of choice when dealing with small renal masses [1]. Laparoscopic partial nephrectomy (LPN) requires exceedingly advanced laparoscopic skills especially during the time-constrained steps of warm ischemia time, such as tumor excision and primary renorrhaphy [2]. With the introduction of the robotic system, robotic assisted partial nephrectomies (RAPN) delivered an unprecedented level of minimally invasive surgery. Its ease of use and faster learning curve exponentially increased adoption amongst practicing urologists around the world [3]. Furthermore, RAPN proved superior to LPN in terms of perioperative outcomes including lower open conversion rates, lower variations in postoperative estimated glomerular filtration rate (eGFR), as well as shorter ischemia time, blood loss, and length of hospital stay [4,5]. During RAPN, the 3-arm and 4-arm approaches have been well described in the literature with the latter being the most commonly used approach, yet there is minimal comparative data amongst the two. We herein describe a comparative experience between the two approaches in terms of surgical parameters, operative marks, and postoperative outcomes.

## Materials and methods

After obtaining institutional review board approval, we retrospectively reviewed our data of all patients who underwent RAPN between August 2013 and December 2017 in a Middle Eastern tertiary referral center, the American University of Beirut. All patients were consented prior to the procedure. Procedures were either fully performed or proctored by a fellowship-trained robotic surgeon, AEH using the da Vinci Si surgical system. The bedside assistant in each case was a junior urology resident with at least two years of experience in laparoscopic surgery (greater than 50 cases). The clinical indication for all cases was a renal lesion suggestive of primary renal malignancy. A comparison study evaluated the differences between a 3-arm robotic approach versus a 4-arm robotic approach in terms of preoperative parameters (demographics, tumor characteristics, tumor complexity), operative marks [estimated blood loss, warm ischemia time, total operating room (OR) time], and postoperative outcomes (length of hospital stay, change in eGFR, complications, margins, pathology results). To note, tumor complexity was determined by the RENAL nephrometry score [6], operative time was measured from the time of the first incision to the last wound closure. Warm ischemia time was measured from the time of arterial occlusion to the last arterial clamp(s) removal. Complications were assessed according to the Clavien-Dindo classification scale [7], whereas the variation in eGFR was calculated by the difference between preoperative creatinine values and the first post-operative creatinine taken at least one month after discharge. 

**Figure 1:**
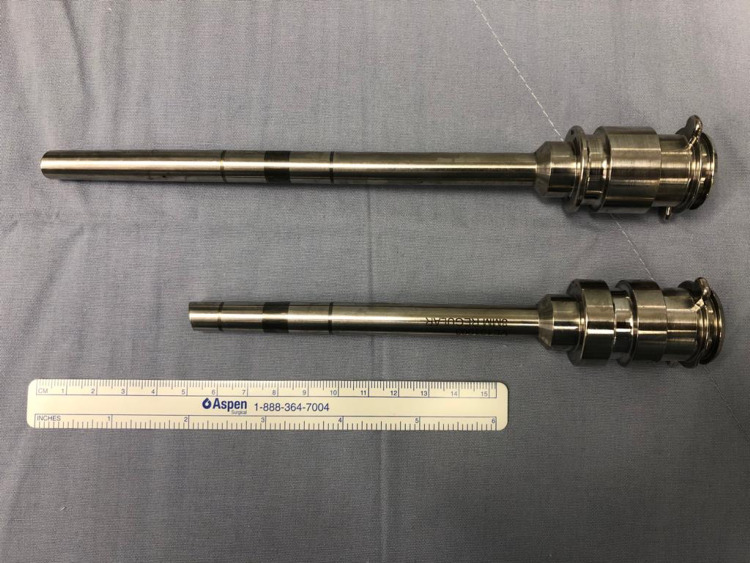
Long port characteristics deployed in a 4-arm approach compared to a traditional port

Statistical methods were performed using the Student’s t-test and chi-squared test. 40 consecutive 3-arm RAPNs and 40 consecutive 4-arm RAPNs were done. All cases were performed via the transperitoneal approach. In all of our 80 cases, two 12-mm trocars were used: one for the camera and the other for bedside assistance. Two standard 8-mm robotic ports were used in the 3-arm technique along with an additional 5-mm assistant port, whereas three 8-mm long ports were deployed in the 4-arm approach. The long ports were characterized by a 16 cm of length and an 8-mm cannula (Figure 1) which aimed at minimizing arm collision. There were no excluded cases in this cohort.

Surgical technique

**Figure 2:**
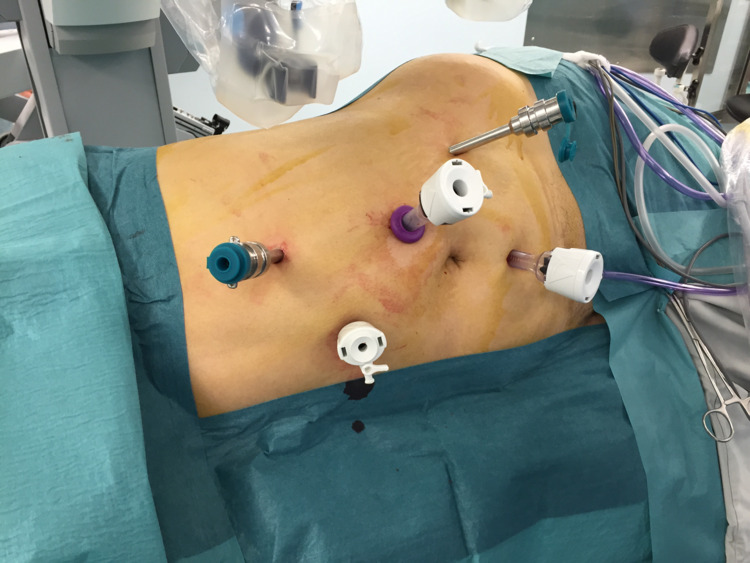
Port placement scheme using a 3-arm approach during a left sided robotic assisted partial nephrectomy

All patients were medically cleared prior to surgical intervention. Preoperative antibiotics were administered within one hour of incision time. General anesthesia was induced and an 18 French Foley catheter was inserted into the bladder. The patient is placed in full lateral position and all bony prominences are appropriately padded. The patient’s body is optimally flexed to ensure maximal flank expansion. A 1 cm pararectus incision is made superior and cephalad to the umbilicus midway between the costal margin and the iliac crest. This is sharply deepened to the rectus fascia accessing the peritoneal space using the Hasson open approach. Pneumoperitoneum is created after insertion of a blunt tip 12-mm trocar. The camera is introduced through this trocar and the peritoneal cavity is inspected. The 12-mm assistant trocar is introduced 2 cm below the umbilicus. With the 3-arm technique, two 8-mm robotic trocars are inserted: one in the subcostal area at the same level of the camera port and another at an equal distance between the camera port and the anterior superior iliac spine (ASIS). An additional 5-mm assistant port is also used. In contrast, with the 4-arm technique, a 3rd robotic trocar (4th robotic arm) is placed at the level of the anterior axillary line 2 cm cephalad to the ASIS with the 4-arm technique. The addition of a 4th robotic arm entails a narrower range of motion, and so an increased chance of intraoperative arm collision. Nevertheless, the risk of collision is minimized by deploying the previously described long ports. Figures 2a and 2b demonstrate the difference in ports’ configuration amongst the two techniques. All ports are inserted under direct vision. The da Vinci robot is then docked to the robotic trocars. Regardless of the technique used, the first step in each of the approaches is adequate exposure between the psoas major muscle and Gerota’s fascia posteriorly. Next, the ureter and gonadal vein are identified and hauled upwards. The renal vein and renal artery are identified and dissected. Upon that, the renal artery is secured by vessel loops and hemlock clips. The Gerota’s fascia is opened and the fat that is surrounding the normal part of the kidney is dissected to delineate the tumor limits. The kidney is completely mobilized for the maximization of tumor access. Tumor location and depth are confirmed by the BK robotic ultrasound probe that portrays onto the console display a picture-on-picture image. Accordingly, the capsular margins of resection are delineated by monopolar cautery. An inventory check of the instruments, gauze, and sutures is routinely performed before clamping of the renal artery and initiation of warm ischemia time. Clamping is performed using the robotic scanlan bulldog. The tumor is excised using cold scissors, avoiding cautery. The deep vasculature on the bed of resection is controlled using running two 3-0 PDS sutures. An outer parenchymal suture using 3-0 Vicryl is then performed using the sliding hemlock technique. The Gerota’s is reapproximated around the renal remnant thus pexing the kidney laterally, the robot is undocked and the trocars are removed under vision. Following that, the specimen is bagged and retrieved via an extension of the camera port incision. The addition of the 4th arm permits optimal exposure during hilar dissection through kidney elevation. In addition, it enhances the surgeon’s control through minimizing assistant dependency during tumor resection.

**Figure 3:**
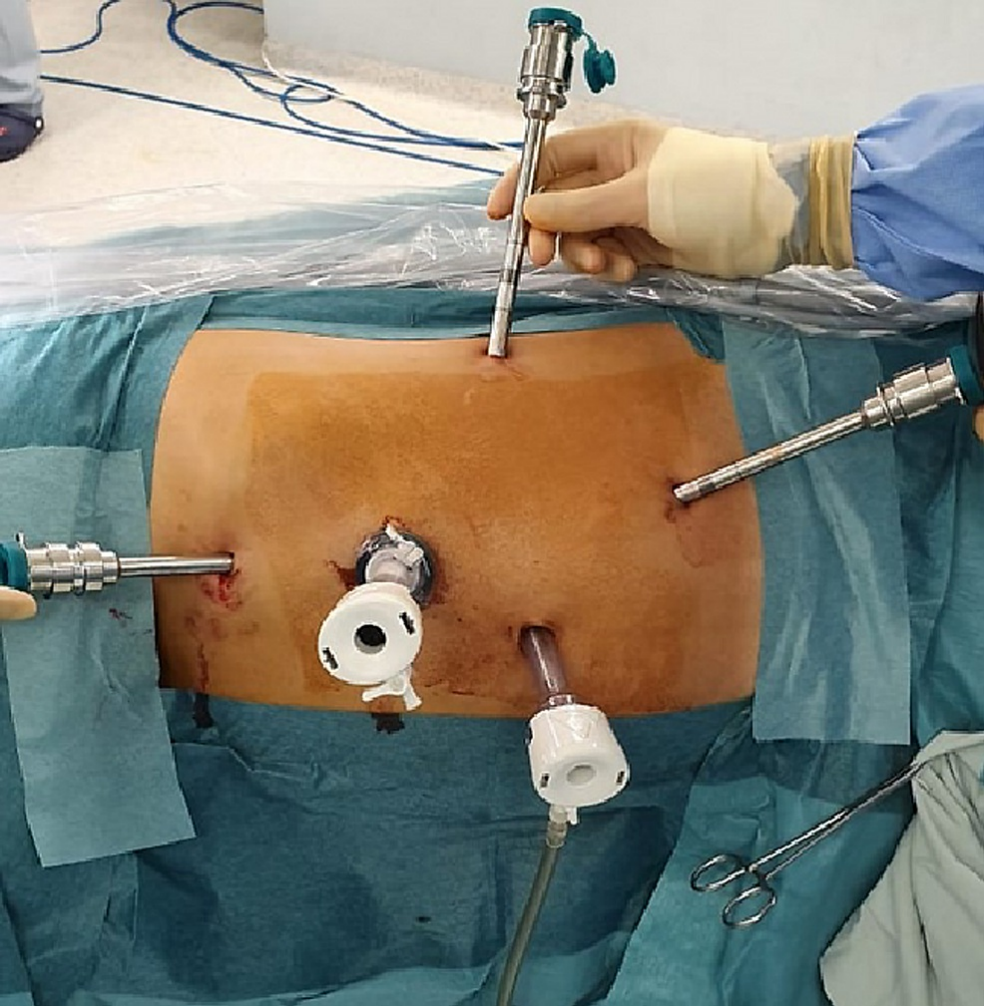
Port placement scheme using a 4-arm approach during a left sided robotic assisted partial nephrectomy

## Results

A total of 40 consecutive 3-arm RAPNs and 40 consecutive 4-arm RAPNs were retrospectively evaluated. The median age and body mass index for the entire cohort was 57 and 30.3 respectively. To note, there was no statistically significant difference among the 3-arm and 4-arm groups in regards to the aforementioned parameters. Differences in tumor complexity (RENAL score) amongst the two groups were statistically insignificant (p-value > 0.05) demonstrated by the mean nephrometry RENAL score and tumor diameter between the 3-arm group and the 4-arm group. The mean nephrometry RENAL score of the 3-arm group was 6.1 compared to 6.9 in the 4-arm group (p=0.19). The mean largest tumor diameter of the 3-arm group was 3.14±1.19 cm compared to 3.44±1.60 cm in the 4-arm group (p=0.35). Table 1 summarizes patient demographics and tumor characteristics of the studied population. Renal cell carcinoma (RCC) was detected on histology in 34 out of 40 cases in the 3-arm group (85%) and in 31 out of 40 cases in the 4-arm group (77.5%). In addition, clear cell renal cell carcinoma was the most common RCC variant among both groups 24 out of 40 cases in the 3-arm group (60%) and 23 out of 40 cases in the 4-arm group (57.5%). 

Perioperative parameters are demonstrated in Table 1, comparing the outcomes of the two techniques. Mean ischemia time was 17.40±6.16 minutes and 17.15±5.35 minutes for the 3-arm and 4-arm groups, respectively (p=0.85). Similarly, surgical outcomes of the two surgical approaches including margin positivity, estimated blood loss, length of hospital stay, and mean change in serum creatinine were similar in the two groups (p-value > 0.05). In contrast, the mean operative time was 42 minutes shorter in the 4-arm group (p=0.01). Post-operative complication rates according to the Clavien-Dindo classification scale were higher in the 4-arm group (p=0.04).

**Table 1:** Tumor characteristics and perioperative parameters

Number of robotic arms=3	Number of robotic arms=3	p-value
Renal score	Low	23(57.5)	13(36.1)	0.192
	Medium	15(37.5)	21(58.3)	
	High	2(5.0)	2(5.6)	
Biggest Size (cm)	Mean (±SD)	3.14±1.19	3.44±1.60	0.351
Pathology	ccRCC	24(60.0)	23(57.5)	
	Papillary RCC Type 1	7(17.5)	2(5.0)	
	Papillary RCC Type 2	1(2.5)	2(5.0)	
	Angiomyolipoma	4(10.0)	0(0.0)	0.024
	Oncocytoma	3(7.5)	7(17.5)	
	Chromophobe RCC	0(0.0)	2(5.0)	
	Other	1(2.5)	4(10.0)	
Ischemia time, (min)	Mean (±SD)	17.40±6.16	17.15±5.35	0.852
Bld loss, (mL)	Mean (±SD)	247.50±135.38	244.36±239.02	0.943
Length of hospital, (days)	Mean (±SD)	3.98±1.05	4.25±1.79	0.405
GFR pre	Mean (±SD)	82.85±22.17	86.175±21.79	0.501
GFR post	Mean (±SD)	72.60±21.34	77.36±23.29	0.346
GFR difference	Mean (±SD)	-10.25±10.09	-8.41±9.22	0.401
Margin	Negative	37(94.9)	37(92.5)	
	Positive	2(5.1)	3(7.5)	1

Table 2 lists the detailed complications seen. None of the RAPNs performed necessitated conversion to open surgery. In addition, none of the cases included in this cohort required ureteral stenting for urinary leaks, whereas two patients required postoperative selective arterial embolization for postoperative bleeding/hematoma. 

**Table 2:** Complications

Number of robotic arms		3	4	p-value
Clavien Dindo Class	0	38(95.0)	30(77.5)	0.041
	1	0(0.0)	3(7.5)	
	2	2(5.0)	4(10.0)	
	3	0(0.0)	2(5)	

## Discussion

Robotic assisted partial nephrectomy (RAPN) has been extensively described over the past years with a progressive emergence of several surgical approaches [8-12]. Prior to the arrival of the Da Vinci Xi platform, the addition of a 4th robotic arm has been debatable [13-16]. Several retrospective studies compared the surgical outcomes between a 3-arm versus 4-arm during RAPN [14-16]. A major drawback related to a 3-arm approach is the universal reliance on an experienced laparoscopic surgical assistant on the bedside; a luxury that is often unavailable. This disadvantage can be obviated by the addition of a 4th arm in RAPN. Furthermore, a 3-arm approach alludes to a more challenging renal exposure as well as an arduous hilar dissection that varies according to the surgeon’s expertise. On the other hand, a 4-arm approach using the da Vinci Si model increases instrument clashing due to an overtly restricted space overcrowded by an additional arm. Theoretically, a 4-arm approach may increase patients’ post-operative incisional pain and may carry a higher risk of port-site hernia.

The release of the da Vinci Xi platform reduced clashing through the ease of port configuration and flexibility of port exchange. As such, a 4-arm technique became the standard approach. Nevertheless, the Si model, the older version of the da Vinci platform, is to date the solely used model in many centers worldwide, hence the need for an approach that reaps the benefits of a 4-arm technique along with minimal arm clashing. As such, the deployment of long bariatric ports rather than the customary ports significantly diminished arm collision. Our results revealed a higher complication rate using a 4-arm approach, whilst small, it is less likely to be related to the addition of the 4th arm, but rather to an increase in tumor complexity of the 4-arm group compared to the 3-arm one with an average RENAL score of 6.9 versus 6.1. On the other hand, ease of hilar dissection, retraction, and surgeon independence instigated a statistically significant decrease in operative time with 4-arm use.

Johnson et al. entertained the opportunity of cost-saving using a 3-arm technique with an estimated amount of $300 per case when compared to a 4-arm technique [14], yet we failed to value their findings given the intraoperative advantage reflected by a decrease in intraoperative surgical time. Consequently, the extra cost is perceived as an acceptable and well-justified trade-off. Furthermore, several studies concluded that in the presence of a well-trained bedside assistant, the addition of a fourth arm for experienced robotic surgeons is unnecessary given the similar operative outcomes between the two techniques [14-16]. As previously mentioned, such commodity is often lacking, as such an additional 4th arm becomes of paramount importance.

This study’s main point of strength is in describing a 4-arm approach in the Si model that enables minimal dependence on an experienced assistant along with diminished collision through careful selection of port characteristics. Furthermore, this is the first Middle Eastern study to present a tertiary referral center’s experience with RAPN performed in the same operative suite reducing operating room variables. 

We acknowledge the limitations of this study. The two compared techniques were retrospectively studied limiting the generalizability of our findings. Moreover, the fact that the 4-arm cohort group was done after the completion of the 3-arm cohort group raises concerns over the attainment of a higher level of surgical expertise at the time of the 4-arm group. In addition, the study was based in an academic institution responsible for surgical training of residents which creates an additional variable to the perioperative parameters. Larger sample size and the involvement of multiple surgeons could potentially buffer such biases. 

## Conclusions

The routine use of a fourth robotic arm during robotic assisted partial nephrectomy offers the operating surgeon a wider exposure and a greater independence during vital steps of surgery. Such an advantage translated to a significant decrease in operative time when using the 4-arm compared to the 3-arm use. Furthermore, use of bariatric ports and standardized trocar placement during the 4-arm approach may decrease arm clashing.
